# 3D ZnO/Ag Surface-Enhanced Raman Scattering on Disposable and Flexible Cardboard Platforms

**DOI:** 10.3390/ma10121351

**Published:** 2017-11-24

**Authors:** Ana Pimentel, Andreia Araújo, Beatriz J. Coelho, Daniela Nunes, Maria J. Oliveira, Manuel J. Mendes, Hugo Águas, Rodrigo Martins, Elvira Fortunato

**Affiliations:** i3N/CENIMAT, Department of Materials Science, Faculty of Science and Technology, Universidade NOVA de Lisboa, Campus de Caparica, 2829-516 Caparica, Portugal; andreiajoiaraujo@hotmail.com (A.A.); bj.coelho@campus.fct.unl.pt (B.J.C.); daniela.gomes@fct.unl.pt (D.N.); mj.oliveira@campus.fct.unl.pt (M.J.O.); mj.mendes@fct.unl.pt (M.J.M.); rm@uninova.pt (R.M.)

**Keywords:** ZnO nanorods, Ag nanoparticles, microwave synthesis, cardboard substrates, SERS

## Abstract

In the present study, zinc oxide (ZnO) nanorods (NRs) with a hexagonal structure have been synthesized via a hydrothermal method assisted by microwave radiation, using specialized cardboard materials as substrates. Cardboard-type substrates are cost-efficient and robust paper-based platforms that can be integrated into several opto-electronic applications for medical diagnostics, analysis and/or quality control devices. This class of substrates also enables highly-sensitive Raman molecular detection, amiable to several different operational environments and target surfaces. The structural characterization of the ZnO NR arrays has been carried out by X-ray diffraction (XRD), scanning electron microscopy (SEM) and optical measurements. The effects of the synthesis time (5–30 min) and temperature (70–130 °C) of the ZnO NR arrays decorated with silver nanoparticles (AgNPs) have been investigated in view of their application for surface-enhanced Raman scattering (SERS) molecular detection. The size and density of the ZnO NRs, as well as those of the AgNPs, are shown to play a central role in the final SERS response. A Raman enhancement factor of 7 × 10^5^ was obtained using rhodamine 6 G (R6G) as the test analyte; a ZnO NR array was produced for only 5 min at 70 °C. This condition presents higher ZnO NR and AgNP densities, thereby increasing the total number of plasmonic “hot-spots”, their volume coverage and the number of analyte molecules that are subject to enhanced sensing.

## 1. Introduction

In the last decade, several efforts have been made to develop inexpensive opto-electronic devices with unique properties, such as flexibility, portability and/or disposability. Several studies have focused their research on paper-based platforms in electronic applications, in order to develop their innovative use as bendable supports, with reliability similar (or even potentially superior) to that of conventional rigid substrates. These paper-based devices are already being used in distinct applications, such as in electronic displays [1], thin-film transistors [2] and solar cells [3], paper batteries [4], ultra violet (UV) sensors [5], biomedical applications [6], and platforms for surface-enhanced Raman scattering (SERS detection) [7,8,9], among others.

The use of a cardboard-type substrate can bring innumerous advantages when compared with other types of flexible substrates, for example, the more common “plastic” materials such as polyethylene naphthalate (PEN), polyethylene terephthalate (PET) and polyimide (PI) [10,11,12]. Among these are the low cost, flexibility, disposability and biodegradability. In addition, the cardboard is mechanically much more robust when compared to common paper substrates. 

On the other hand, zinc oxide (ZnO) is a promising multifunctional, n-type semiconductor material, with a wide and direct band gap of about 3.37 eV and a large free exciton binding energy of 60 meV at room temperature [13]. This material is also biocompatible and displays piezoelectric properties. Because of these different properties, ZnO is attractive for various bio-, micro- and nano-electronic applications, such as in thin-film transistors [14], transparent conductive oxides [15,16,17,18], dye-sensitized [19] and perovskite [20] solar cells, UV/ozone sensors [21,22], piezoelectric devices [23], glucose sensors [24] in biomedical science as antibacterial and antifungal agents [25] and as a platform for SERS detection [26,27,28].

Many efforts have been devoted to obtaining ZnO nanostructures with enhanced optical and electrical properties while maintaining good chemical stability. For this propose, different growth techniques, precursors and solvents are continuously being investigated to prepare a variety of ZnO nanostructures, such as chemical vapour deposition [29], electro-deposition [30], electro-spinning [31], laser-assisted flow deposition (LAFD) [32] and hydrothermal methods either by conventional heating or assisted by microwave radiation [5,33].

The use of hydrothermal synthesis assisted by microwave radiation in the growth of ZnO nanorods (NRs) has the advantage of providing a short reaction time and thus lower energy consumption, enhanced reaction selectivity (dependent on the solvents and precursors used) and a homogeneous volumetric heating with a high reaction rate [33,34]. With this technique, there are many factors that can influence the growth of ZnO NRs, such as the precursors/solvents used and their concentration, the synthesis time and temperature, the type of seed layer and the substrate used [35,36,37,38,39].

The application of microwave-assisted synthesis in the direct growth of ZnO nanostructures on paper-based substrates is still scarce in the literature; only a few reports exist on applications that usually require some hours to grow this type of nanostructure [40,41]. In contrast, in this work, ZnO nanostructures are grown in a few minutes. The structural and optical properties of ZnO nanostructures obtained by hydrothermal synthesis assisted by microwave radiation and grown on cardboard substrate have been analyzed here by changing the time and synthesis temperature.

To the best of our knowledge, this is the first report demonstrating the direct growth of ZnO NRs on cardboard substrates using a low-cost and ultra-fast synthesis method (requiring a few minutes) via microwave-assisted hydrothermal synthesis. After complete characterization, these structures were tested as platforms for SERS application by evaporating an ultra-thin silver (Ag) film on the ZnO NRs and allowing the formation of Ag nanoparticles (AgNPs) by a dewetting method [7,42].

When metal nanoparticles are excited by electromagnetic radiation, their free electrons collectively oscillate, resulting in a localized surface plasmon resonance (LSPR) [43,44,45]. SERS is a technique that allows highly sensitive structural detection of low-concentration analytes through the amplification of the electromagnetic field generated by the excitation of localized surface plasmons [7,8,46]. The strong enhancement of the local electric near-field intensity in the vicinity of the metallic nanoparticles sustaining LSPRs can highly amplify the Raman scattering signals close to the particles’ surface. Thus, a much more pronounced Raman scattered signal at the surface can be detected when molecules are adsorbed onto the metallic nanoparticles [47,48].

This localized enhancement effect allows the detecting of extremely small amounts of analytes, making SERS an efficient tool for the detection of a variety of problems, including corrosion, detection of chemical warfare agents, bacteria on food, trace evidence in forensic science, and blood glucose, among others [49,50,51,52].

A tremendous amount of work on the fabrication of various cost-efficient cellulose substrates for SERS has already been performed by various research groups [7,53,54,55,56]. These types of substrates can have Raman signal enhancements (enhancement factor—EF: 10^5^–10^7^) comparable with the conventional rigid and planar supports such as glass, silicon wafers and aluminium films. Nevertheless, paper substrates for SERS have several advantages over conventional rigid substrates in terms of cost, flexibility, portability, eco-friendliness and biodegradability. For instance, such substrates are able to collect analytes when used in contact with the human body or food in packaging [57,58], as they can be wrapped around curved surfaces, opening doors for the next generation of biomedical optical sensing.

Three-dimensional (3D) SERS substrates with different morphologies, such as NRs, nanotubes and nanowires, have been proposed as promising SERS substrates [8,26,59,60]. In all of these cases, SERS activity is due to the presence of metal nanoparticles such as gold (Au) or Ag. Among the various materials and morphologies, ZnO NRs have been considered the preferential candidates for the fabrication of SERS substrates because of their high surface-to-volume-ratio morphology and ease of fabrication, employing inexpensive and fast growth methods [8,61]. The research on 3D hybrid substrates is relatively new, and it is quickly moving towards the fabrication of 3D hybrid structures on low-cost bendable substrates [8].

In this work, a simple and scalable two-step method is presented. ZnO NRs are grown on a cardboard substrate using a low temperature (70 to 130 °C) and reasonably fast hydrothermal method assisted by microwave radiation (5 to 30 min), followed by a uniform and large-scale method to deposit metal nanoparticles on cellulose-based substrates on the basis of thermal evaporation assisted by an electron beam.

Up to now, little has been known about the influence of 3D ZnO NRs structures (width and length) on the morphology of the nanoparticle structures formed on top and, consequently, on the detected SERS signal. Here, it will be demonstrated how the resulting ZnO NR arrays can influence and serve as an effective SERS platform, allowing a strong SERS enhancement factor and enabling the detection of rhodamine 6 G (R6G) at the low concentration of 10^−6^ M. Thereby this shows that the produced ZnO NRs on cardboard-type substrates can be used as a low-cost, disposable and highly sensitive SERS platform for the detection of biomarkers for distinct medical diagnostic applications [62,63]. Moreover, the approach developed in this work, besides its application in SERS, can also be used in photocatalysis, in gas sensors and as an antimicrobial assay. In fact, several reports have already demonstrated different applications for ZnO nanostructures decorated with Ag, demonstrating it to be a versatile material [64,65,66].

## 2. Results and Discussion

### 2.1. Characterization of Cardboard Substrate: Thermal Analysis and X-ray Diffraction

The cardboard packaging substrates used in this study consist of several compressed layers of cellulose fibres, polymeric coatings (polyethylene) and evaporated aluminium (see Figure S1c in Supplementary Information). The use of a polymeric coating on this type of cardboard has two major purposes: one is to promote the proper adhesion of the metalized layer to the cellulose, and the other is to protect the thin metalized aluminium layer from scraping or from other environmental damage. A thin native oxide (Al_x_O_y_) layer is also present on top of the aluminium layer [7].

The morphology and surface roughness of this type of substrate have been previously studied by the authors [7], revealing both a larger surface roughness in the micrometre range and smaller nanoscale features with a root mean square (RMS) roughness of 2.37 (±0.05) nm, as determined by AFM (Atomic force microscope).

In order to understand the robustness of this type of substrate when subjected to higher temperatures, such as during the microwave-assisted hydrothermal synthesis, differential scanning calorimetric (DSC) and thermo-gravimetric (TG) measurements were carried out (see Figure S1a in Supplementary Information). The DSC curve presents a very small endothermic peak at 105 °C, accompanied by a small weight loss (about 5%), which is characteristic of the desorption or drying of cellulose fibres. At 350 °C, another endothermic peak is observed, accompanied by a large weight loss (of about 60%), which can be correlated with the decomposition of cellulose fibres [67].

Therefore, these results ensure that the substrate can be heated up to 220 °C without damage and without losing its properties, that is, up to the temperature at which the mass of the sample starts to decrease, indicating the maximum working temperature for this type of substrate. 

The X-ray diffraction (XRD) diffractogram obtained for the cardboard substrate reveals the peaks of cellulosic fibres, corresponding to the crystallographic planes (110), (200) and (004), respectively, which are in accordance with those reported in the literature [2,68] (see Figure S1b in Supplementary Information). The peaks corresponding to the aluminium crystallographic phase, associated to the crystallographic planes (200), (220) and (311), respectively, can also be observed [69]. No other crystalline phases were detected.

As a result of the presence of these very intense peaks from the cellulosic fibres and aluminium layer, the XRD analysis of the ZnO NRs’ crystallographic phase was performed only between 30° and 38°.

### 2.2. Morphology and Crystallographic Structure of ZnO Nanorods

To infer the morphology and the crystallographic structure of the synthesized materials, scanning electron microscopy (SEM) and XRD experiments were carried out on all the produced samples. The synthesis time and temperature are two of the most important parameters in ZnO NR synthesis that control the resulting morphology.

**Figure 1:**
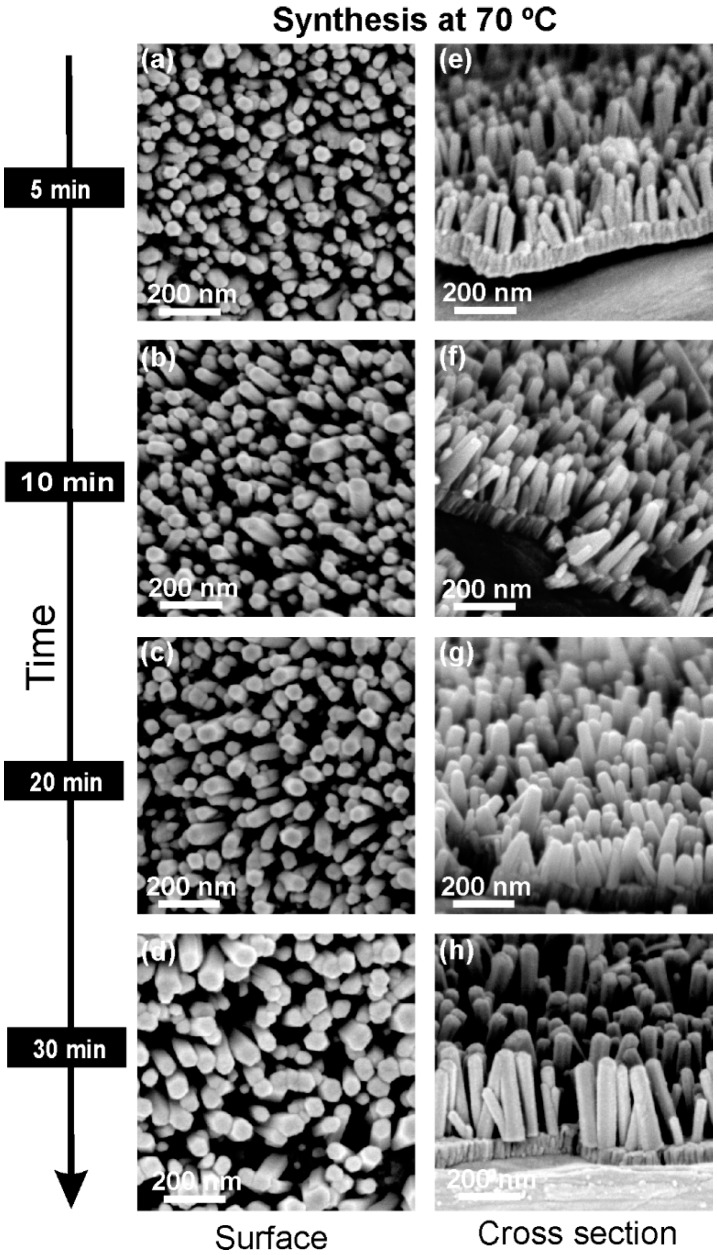
Surface and cross-section scanning electron microscopy (SEM) images of ZnO nanorods produced by the hydrothermal method assisted by microwave radiation at 70 °C, with different synthesis times on cardboard substrate: (**a**,**e**) 5 min; (**b**,**f**) 10 min; (**c**,**g**) 20 min; (**d**,**h**) 30 min.

SEM analysis for samples produced at 70 °C for different periods of time (5, 10, 20 and 30 min) are presented in Figure 1. It is noteworthy that the NRs’ shape is hexagonal regardless of the synthesis time. Nevertheless, with the increase in the synthesis time, it is possible to observe that the ZnO NRs become wider, with a less compact distribution and widths ranging from 45 (±5), 50 (±5), and 55 (±5) to 75 (±5) nm for synthesis times of 5, 10, 20 and 30 min, respectively. From the cross-sectional SEM images of Figure 1, it is clear that the ZnO NRs’ growth is “quasi-aligned” vertically from the substrate. The NRs’ length also increases with a longer time, presenting values of approximately 150 (±5), 175 (±10), 200 (±20) and 325 (±20) nm for synthesis times of 5, 10, 20 and 30 min, respectively. These results are consistent with those reported in the literature [35]. The size of the ZnO NRs depends on the concentration of [Zn^2+^] ions in the solution. Thus, considering the initial solution concentration, the concentration of ions in the solution will decrease with the increase in the particle size. Therefore, a longer synthesis time implies prolonged NR growth, which occurs with a constant crystal growth rate that allows the formation of NRs with a constant diameter from the bottom to the top, as for those observed in the cross-section images of Figure 1. As such, a longer synthesis time will add more ions to the ZnO NRs’ crystallographic structure and make them grow in length and width, while maintaining the rods’ alignment.

**Figure 2:**
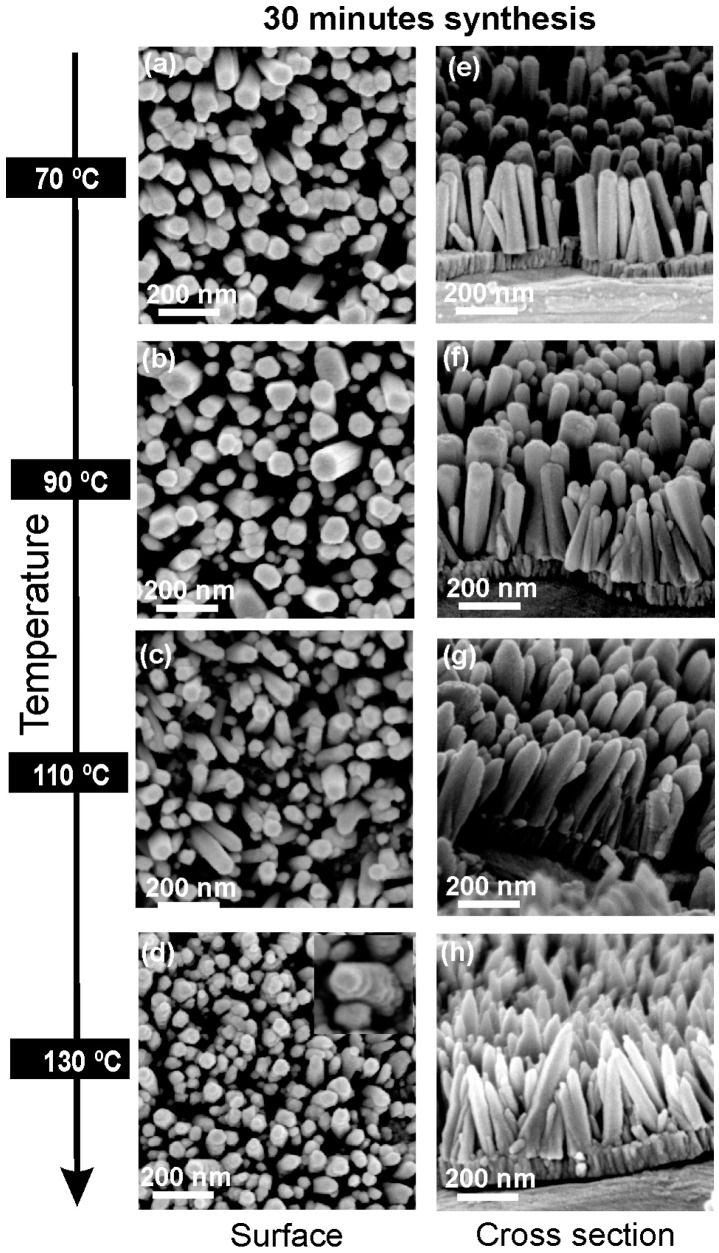
Surface and cross-section scanning electron microscopy (SEM) images of ZnO nanorods produced by hydrothermal method assisted by microwave radiation for 30 min, with different synthesis temperatures on cardboard substrate: (**a**,**e**) 70 °C; (**b**,**f**) 90 °C; (**c**,**g**) 110 °C; (**d**,**h**) 130 °C.

Figure 2 shows the SEM analysis of samples produced with a different temperature (70, 90, 110 and 130 °C) for 30 min. With the increase in the synthesis temperature, it is interesting to observe that the ZnO NRs become wider and then thinner again. The NRs’ diameter increases from approximately 70 (±5) nm (at 70 °C) to 120 (±20) nm (at 90 °C), decreasing to 80 (±5) nm (at 110 °C) and then to 50 (±5) nm (at 130 °C). This may be due to the fact that the microwave power needs to be turned on more frequently during the synthesis in order to maintain the higher temperatures [34]. Regarding the NRs’ length, this increases monotonously from 300 (±20) nm to 500 (±20) nm with the increase in temperature, in accordance with literature studies [70]. Additionally, with the increase in the synthesis temperature, the ZnO NRs become more misaligned and the top of the NRs becomes sharper. With the increase in temperature, the NRs’ top changes from a flat to a pencil-like shape. This may be related with anisotropy in the growth-rate direction, caused by the increase in temperature. In the hydrothermal synthesis of ZnO, it has been reported by some authors that the higher crystal growth velocity is in the [0001] direction [71]. As such, the appearance of the pencil-like NR structure suggests that the growth rate of the (001) crystal facet is relatively faster at a higher temperature when compared with the NRs with a flat top, produced at lower temperature [71,72].

**Figure 3:**
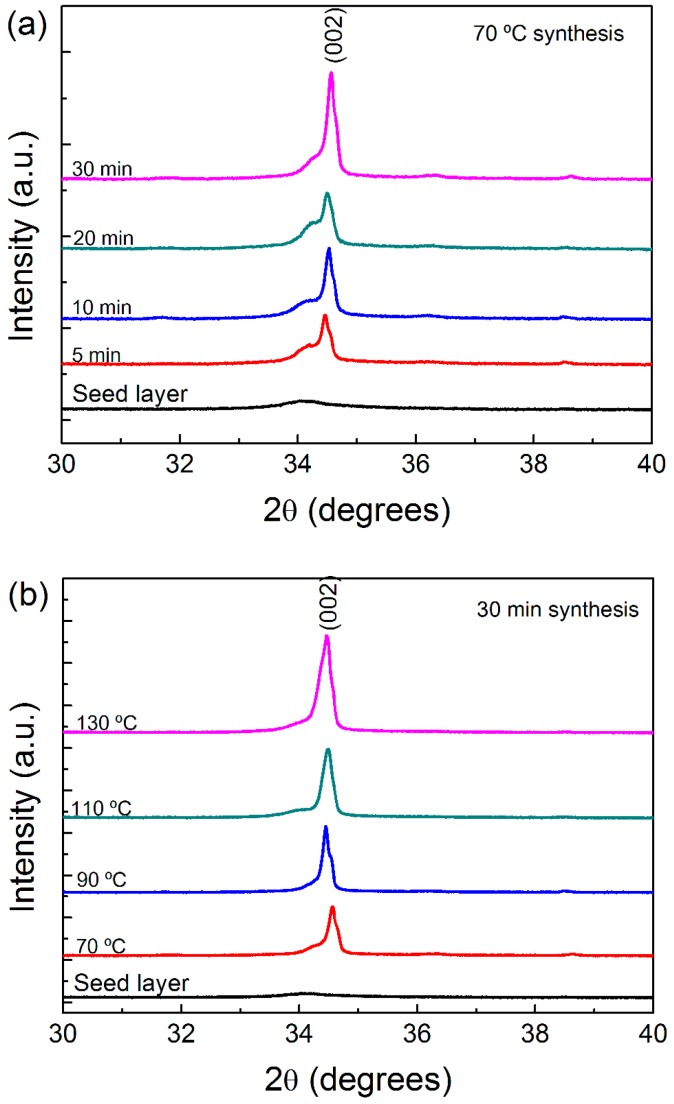
X-ray diffraction (XR)D diffractograms of ZnO nanorod arrays produced by the hydrothermal method assisted by microwave radiation: (**a**) with a temperature of 70 °C for 5, 10, 20 and 30 min; (**b**) for 30 min with a temperature variation between 70 and 120 °C.

The XRD diffractograms of the produced ZnO NR arrays grown on a cardboard substrate are presented in Figure 3. All the samples presented similar XRD patterns, indicating that the ZnO NRs possess a high crystallinity. It is possible to observe that, for all the produced samples, a single peak at 2θ = 34.4° is present, being fully assigned to the (002) plane of the hexagonal wurtzite ZnO structure and displaying lattice constants of *a* = 0.3296 nm and *c* = 0.5207 nm, in accordance with [13]. This result shows that the ZnO NRs are well oriented along the *c*-axis direction.

**Figure 4:**
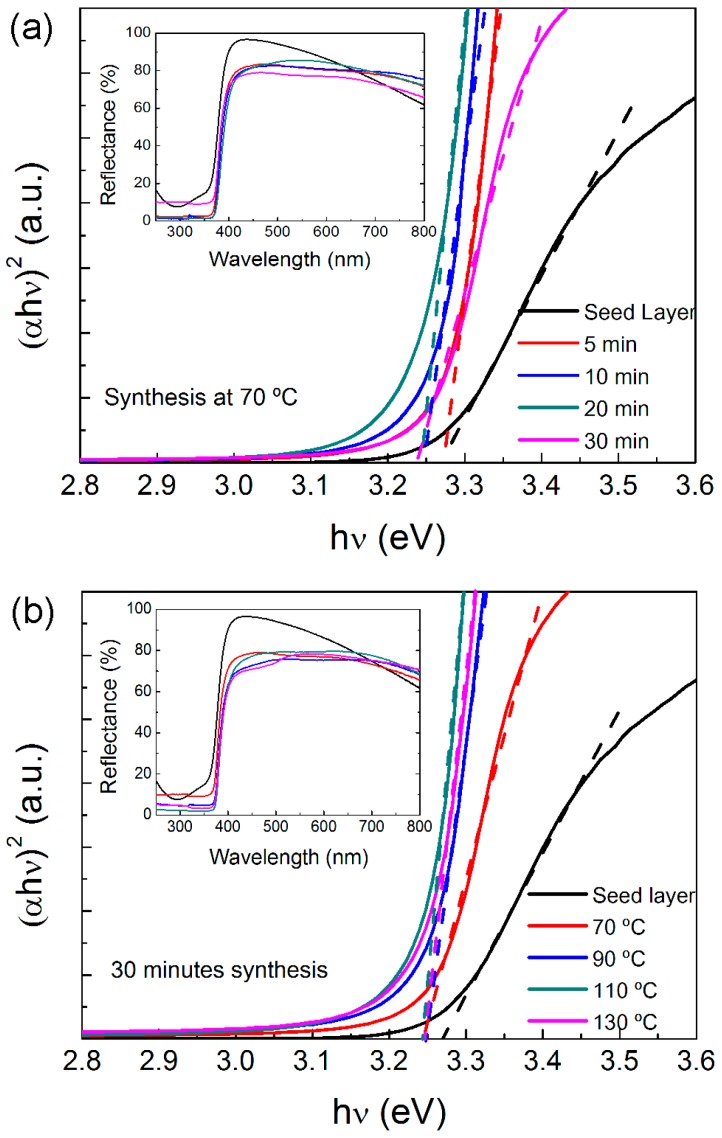
Tauc plots used for the determination of the optical band gap of the ZnO nanorod arrays produced by the hydrothermal method assisted by microwave radiation, on cardboard substrate: (**a**) with a temperature of 70 °C for 5, 10, 20 and 30 min; (**b**) for 30 min with a temperature variation between 70 and 130 °C.

For synthesis at 70 °C with different process times (see Figure 4a), the peak intensity increases with the increase of the synthesis time, which can be attributed to the increase in the grain size [73] or to the increase in the NRs’ length (as confirmed by the SEM images of Figure 1). A larger rod width will also increase the XRD signal.

With the increase in the synthesis temperature, there is an increase in the intensity of the (002) peak, which suggests that the ZnO NRs become more crystalline. This can be attributed to a change in the crystal size of the ZnO material along the rods (see Figure 3b) [73]. Additionally, it is possible to see that, for lower synthesis temperatures, a broad peak still appears, which corresponds to the XRD of the seed layer. This bump disappears with the increase in the synthesis temperature, likely as a result of the increase in the NRs’ size. At higher synthesis temperatures, other ZnO crystallographic peaks begin to appear (even if they are almost imperceptible), which indicates that the NRs begin to become more misaligned. These results confirm that pure and quasi-aligned ZnO nanostructures were obtained by microwave-assisted synthesis on cardboard substrates, in accordance with observations in the SEM micrographs of Figure 1 and Figure 2.

### 2.3. Optical Properties

The optical band gap of the ZnO NR structures, produced with a different synthesis temperature and time on the cardboard substrates, was evaluated from the reflectance spectra shown in the insets of Figure 4. The aluminium coating on such substrates (see Figure S1a in Supplementary Information) acts as a mirror, reflecting most of the impinging light. Therefore, the decrease in reflection is mainly given by light absorption occurring in the ZnO NRs’ material. As such, its band gap can be determined through the Tauc equation for direct band semiconductors [74]:

(1) $${\left(\alpha h\nu \right)}^{m\;}=A\left(h\nu -\;E_{g}\right)$$

where *α* is the material absorption coefficient, *h* is the Plank constant, *ν* is the frequency, *m* is a constant that depends on the type of optical transition (i.e., *m* = 1/2 for allowed direct transitions and *m* = 2 for allowed indirect transitions), *A* is a photon energy-independent constant and *E_g_* is the material optical band gap.

Figure 4 shows the optical bandgap calculated by extrapolating (*αhν*)^2^ versus *hν*. It is possible to observe that, in general, the ZnO NRs absorb almost all light in the UV region, as the material reflectance almost decays to zero. The higher reflectance in the visible range, observed for the sample with only the ZnO seed layer, is due to the low thickness of the layer (100 nm) and to the small sputtered ZnO grains that absorb less radiation.

The estimated optical band gap values are indicated in Table 1. The band gap decreases with the increase in the synthesis time, changing from 3.27 to 3.24 eV. However, with the increase in the synthesis temperature, the band gap value remains constant at 3.24 eV.

It is well known that the optical band gap of a semiconductor depends on distinct parameters, such as the residual strain, crystal defects, impurities and grain-size confinement [75]. Moreover, the band-gap value usually decreases with the increase in grain size and NR length.

### 2.4. Decoration of Ag Nanoparticles on ZnO Nanorods for SERS Platforms on Cardboard

**Figure 5:**
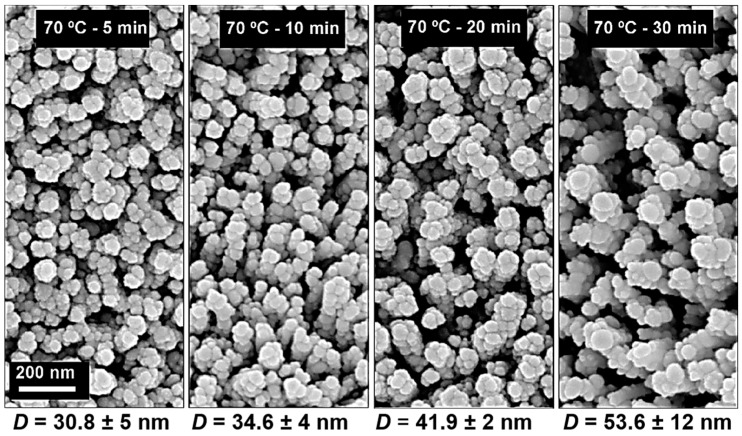
Scanning electron microscopy (SEM) images of ZnO nanorods (NRs) with Ag nanoparticles deposited by thermal evaporation. The NRs were produced at 70 °C with different synthesis times (5, 10, 20 and 30 min). The corresponding values of the average NP size (in-plane major axis, *D*) of the Ag NPs deposited on the nanorods, from 6 nm mass equivalent thicknesses, are indicated below the images.

The ZnO NRs need to be decorated with metal nanoparticles to exhibit SERS activity; thus the NRs were coated with Ag nanoparticles deposited by thermal evaporation (Ag NPs@ZnO NRs). As previously reported [7], the desirable particle sizes for molecular SERS detection correspond to those formed with a 6 nm Ag mass-equivalent film thickness, resulting in a uniform array of NPs with an average long-axis diameter of around 60 nm. Figure 5 shows the variation of the Ag NP structures, formed from such a 6 nm mass thickness, when deposited on ZnO NRs produced at 70 °C with different synthesis times.

Close-packed Ag NPs were distributed uniformly on the top and sidewalls of the hexagonal ZnO NRs, leading to a large surface-to-volume ratio of the plasmonic nanostructure. The average Ag NP diameter (*D*) was measured for all samples (indicated in Figure 5), and it was found that this decreases when decreasing the NR synthesis time. Such a decrease is due to the lower NR width (and thus lower sidewall area; see Figure 2), which hinders the coalescence of the Ag NPs during their formation [42]. Nonetheless, the average separation between adjacent Ag NPs remains similar (less than 5 nm) for all cases of Figure 5, which is critical to create high SERS activity within their interspace excited by the surface plasmon coupling between the narrow gaps (called “hot spots”) of neighbouring NPs [76,77].

**Figure 6:**
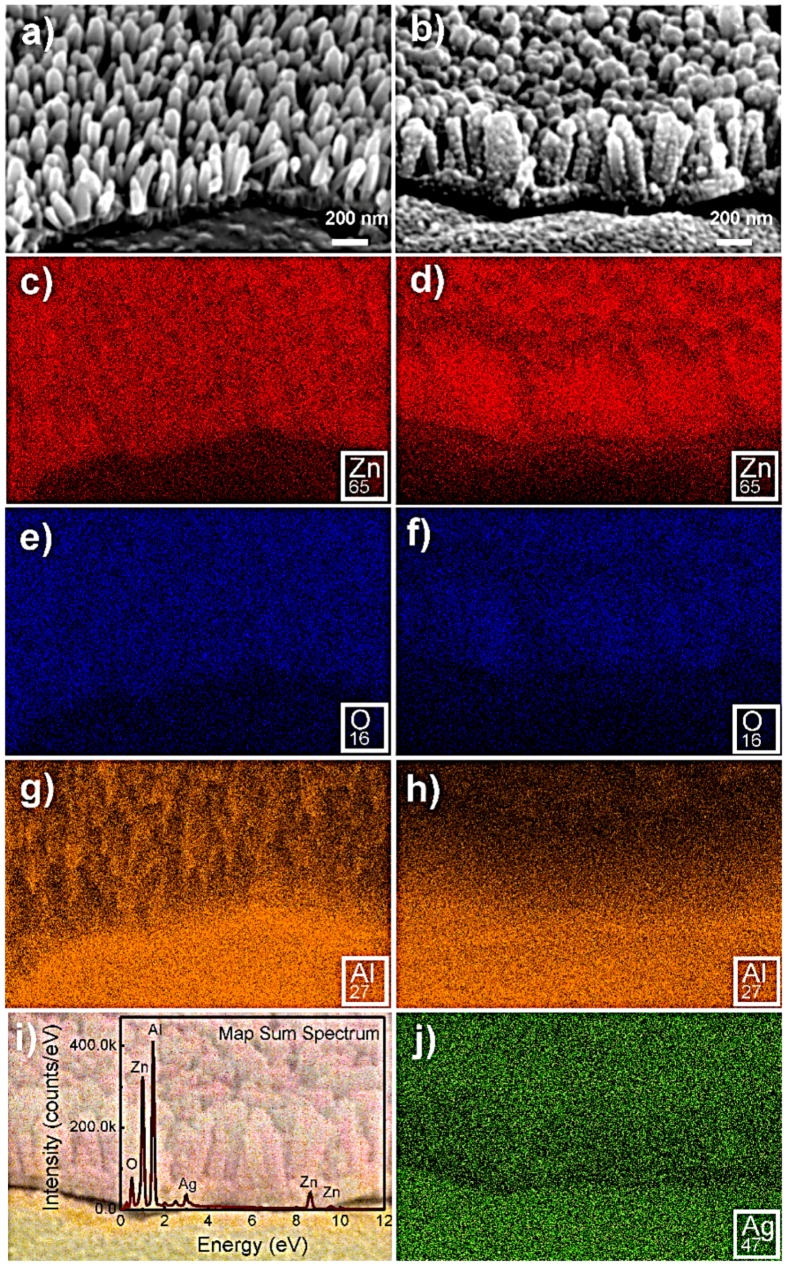
Scanning electron microscopy (SEM) images of ZnO nanorods (NRs) formed at 70 °C with a synthesis time of 5 min (**a**) and ZnO NRs covered with silver nanoparticles (Ag NPs) (**b**). The respective X-ray maps corresponding to Zn are presented in (**c**) and (**d**), O in (**e**) and (**f**), Al in (**g**) and (**h**), and Ag in (**j**). (**i**) Map sum spectrum of the sample with Ag NPs covering the ZnO NRs.

Energy dispersive spectrometer (EDS) mapping has been carried out and the results are presented in Figure 6 for the ZnO samples produced at 70 °C with a synthesis time of 5 min. The EDS analyses attested to the homogeneous distribution of Ag NPs, covering completely the ZnO NRs’ surface.

**Figure 7:**
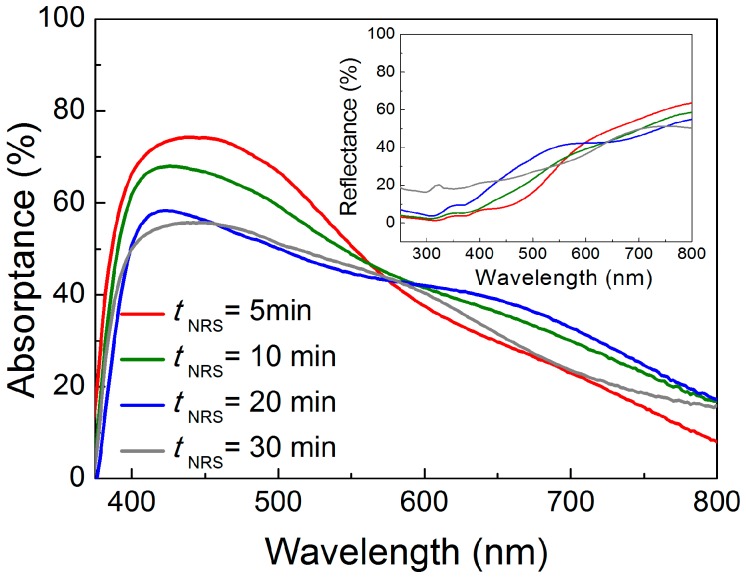
UV-Vis-NIR (ultra violet - visible - near infrared) absorption spectra of the ZnO nanorod substrates produced at 70 °C with different synthesis times (5, 10, 20 and 30 min), after silver nanoparticle deposition with 6 nm Ag mass equivalent thickness.

To study the LSPR spectral positions of the SERS substrates, the NP absorptance spectra, *Abs* (Figure 7), determined from the total reflectance, *R_T_*, spectra before and after the Ag NPs’ deposition, was calculated using the following equation:

*Abs* = *R_T_* (Substrate + ZnO NRs) − *R_T_* (Substrate + ZnO NRs + NPs) (2)

It should be pointed out that the spectrophotometry measurements of Figure 7 can only detect the far-field light extinction caused by the particles, and they do not probe their near-field light scattering, which is responsible for SERS. Nevertheless, the analysis of such spectra is important to determine the spectral location and extension of the LSPRs and, thereby, to enable matching with the wavelengths of the incident laser and Raman-scattered photons.

**Figure 8:**
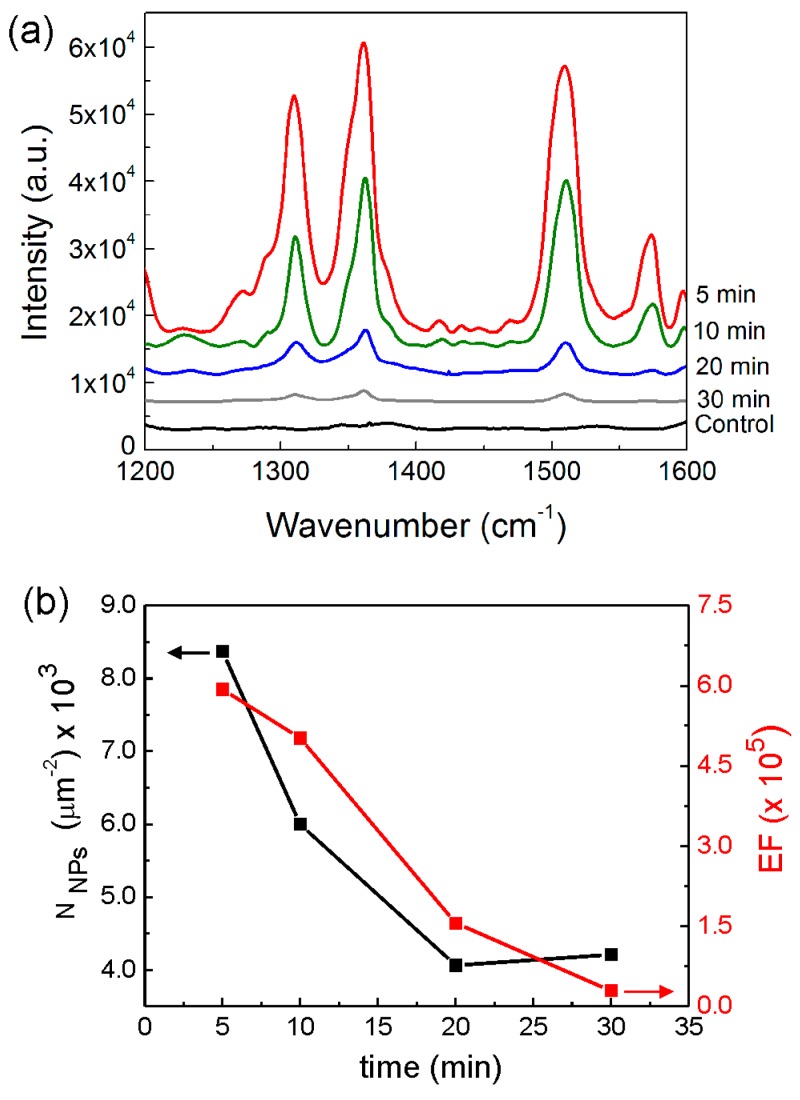
(**a**) Raman spectra of 10^−6^ M of rhodamine 6 G (R6G) on ZnO nanorods (NRs) with and without (control) Ag nanoparticles (NPs). The NRs were produced at 70 °C with different synthesis times (5, 10, 20 and 30 min). (**b**) Enhancement factor (EF) and estimated number of Ag NPs (N_NPS_) per square micrometre, as a function of the ZnO NR synthesis times.

Figure 8a shows that the intensity of the Raman signal (using 10^−6^ M R6G analyte solution) is pronouncedly amplified when the NR synthesis time (*t*_NR_) decreases from 30 to 5 min. We also provide an estimation of the SERS EF achieved in each sample, calculated according to the following expression [78,79]:

(3) $$EF=\frac{I_{SERS}}{I_{Raman}}\times \frac{N_{Raman}}{N_{SERS}}$$

where *I_SERS_* is the SERS intensity of a specific Raman vibrational line of the R6G spectrum, *I_Raman_* is the normal R6G Raman intensity analysed over a non-plasmonic reference substrate (glass). *N_Raman_* and *N_SERS_* are, respectively, the estimated average number of adsorbed molecules that produce the Raman reference and SERS signals [78]. In the present measurements, *N_SERS_* corresponds to the estimated number of molecules adsorbed on the surface contributing to the SERS signal, while *N_Raman_* is the total number of molecules contributing to the reference Raman signal (from non-SERS substrate). Both values are related with the laser spot focus, and they are determined by the relation:

(4) $$N_{SERS\;}=\;\eta \;\times \;N_{A\;}\times V\;\times \;C_{SERS}\frac{A_{Laser}}{A_{SERS}}$$

(5) $$N_{Raman}=\;N_{A}\;\times V\;\times \;C_{Raman}\;\times \;\frac{A_{Laser}}{A_{Raman}}$$

where *N_A_* is the Avogadro number, *V* is total volume of the analyte solution drop spread on the substrate (2 μL), *A_Laser_* is the area of the laser spot (8.32 × 10^−7^ mm^2^) projected in the horizontal plane, and *A_SERS_* and *A_Raman_* are the areas of the solution drops on the SERS and non-SERS reference substrate respectively. *C_SERS_* and *C_Raman_* are the concentrations of analyte solutions applied over the SERS and non-SERS substrates, respectively. Because the same volume of the solution was applied in both substrates and their surfaces had similar hydrophilicity (determining the drop spreading), it is possible to assume that *A_SERS_ ≈ A_Raman_* = 3.14 mm^2^. The dimensionless adsorption factor on the SERS substrate, *η*, was taken to be 0.3 in accordance with previous reports that use similar types of substrates [80]. This adsorption factor is based on the Langmuir isotherm and can be expressed in the form *η* = 1/(1 + K*c*_0_), where *c*_0_ is the initial concentration of the analyte at saturation level and *K* the equilibrium binding constant. For the determination of the EF (Figure 8b), the calculations of the intensities consider the area under the Raman vibrational lines at 1360 cm^−1^.

The average EF obtained with the *t*_NR_ = 30 min substrate was 3 × 10^4^, while with the *t*_NR_ = 5 min substrate, an average enhancement of 7.0 × 10^5^ was achieved. The observed trend of higher EF with a lower NR synthesis time is chiefly attributed to the higher number of Ag NPs present per unit area of the substrate surface. Figure 8b presents a correlation between the EF variation and the estimated number of nanoparticles (N_NPS_) per square micrometre, as a function of the different ZnO synthesis times.

The observed highest SERS enhancement of the *t*_NR_ = 5 min substrate can be ascribed to the higher estimated nanoparticle density on the sample, as a result of the increase in the NR density (described in Section 2.2), providing an increase in the total number of plasmonic hot-spots and an extended area for the deposition of analyte molecules covered by such regions of strong near-field intensity [8,26,81]. 

**Figure 9:**
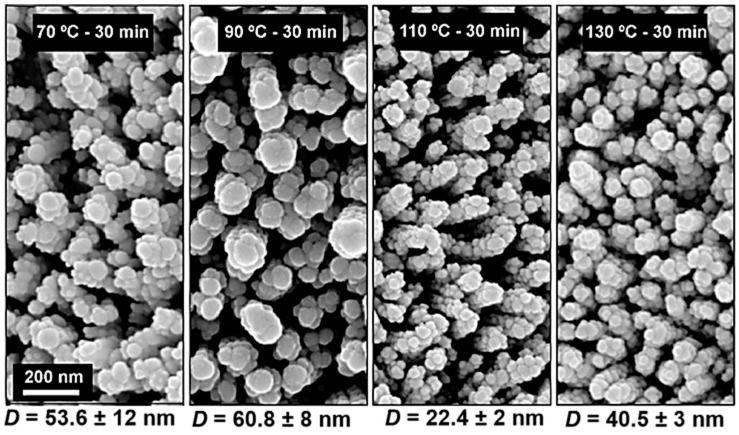
Scanning electron microscopy (SEM) images of ZnO nanorods (NRs) with Ag nanoparticles (NPs) deposited by thermal evaporation. The NRs were produced over 30 min with different synthesis temperatures (70, 90, 110 and 130 °C). The values for the average size (in-plane major axis, *D*) of the deposited Ag NPs from 6 nm mass thicknesses are indicated.

In Figure 9, it is possible to observe the variation of Ag NPs deposited on ZnO NRs produced over 30 min with different synthesis temperatures varying from 70 to 130 °C. In this case, the Ag NPs presented different diameters, which approximately followed the trend of the NRs’ average width shown in Figure 2. However, despite the fact that the morphology, alignment and density of the NRs changed considerably for this set of samples, the SERS signals obtained were similar (with EF of ~10^5^), regardless of the different NR synthesis temperatures. This may be due to the fact that, for higher temperatures, the ZnO NRs’ density decreases and the top of the NRs change from flat to pencil-like tops, leading to the decrease in the number of hot-spots on the NRs’ upper illuminated part. On the other hand, because at higher temperatures the NRs became misaligned (as previously reported), the unpolarized illuminating electric field incident from the top becomes more aligned with the longest NPs’ axes along their base diameter, thus allowing higher electromagnetic enhancement than for the vertically aligned NRs. This can compensate for the disadvantageous pencil geometry of the NRs formed at a higher temperature and contribute to keeping the SERS signal approximately unchanged among the structures of Figure 9.

## 3. Experimental Details

### 3.1. Synthesis of ZnO Nanostructures

**Figure 10:**
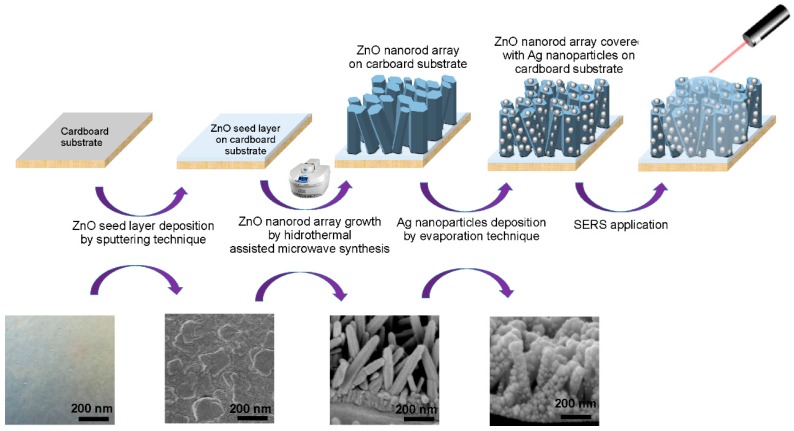
Schematic of the production process of ZnO nanorod arrays by hydrothermal synthesis assisted by microwave radiation and decorated with Ag nanoparticles, on cardboard substrates, for surface-enhanced Raman scattering (SERS) applications.

The ZnO NR arrays have been synthesized by a hydrothermal method assisted by microwave radiation on cardboard substrates coated with a ZnO seed thin film (see Figure 10).

The ZnO seed layer was deposited on the cardboard substrate by room-temperature radio frequency (RF) sputtering. A ceramic ZnO oxide target with a purity of 99.99% was used for the deposition. Prior to the depositions, the chamber was evacuated to a base pressure of 10^−6^ mbar. A shutter between the target and the substrate enabled the protection of the targets from cross-contamination. Regarding the ZnO seed layer deposition conditions, a power density of 12.30 W·cm^−2^ and a deposition pressure of 4 × 10^−3^ mbar were used. The distance between the target and substrate was fixed at 15 cm. The deposition time was 60 min, allowing for the formation of a layer with a 100 nm thickness.

After uniformly coating the cardboard substrates with the ZnO seed layer, ZnO NR arrays were synthesized by the hydrothermal method assisted by microwave radiation, using the microwave system Discover SP, from CEM (Matthews, NC, USA). The ZnO seeded substrates (20 × 20 mm) were placed at an angle against the Pyrex vessel, with the seed layer facing down [82], and were filled with an aqueous solution of 25 mM zinc nitrate hexahydrate (Zn(NO_3_)_2_·6H_2_O; 98%, CAS: 10196-18-6) and 25 mM hexamethylenetetramine (C_6_H_12_N_4_; 99%, CAS: 100-97-0), both from Sigma Aldrich (St. Louis, MO, USA).

Two sets of experimental conditions were tested for a microwave power input of 50 W: (1) The variation of the synthesis time over 5, 10, 20 and 30 min with a constant temperature of 70 °C, which is the minimum temperature required for ZnO NR formation. (2) The variation of the temperature over 70, 90, 110 and 130 °C, employing the longer 30 min synthesis time in order to have a higher width/length ratio of the NRs. After each synthesis process, the materials were cleaned with deionized water and isopropyl alcohol and were dried with compressed air.

After the growth of ZnO NRs on the cardboard substrates, Ag NPs were deposited using an electron gun-assisted thermal evaporation technique [42] to be able to use the substrates as platforms for SERS. The temperature during the evaporation process was held at 150 °C and the Ag layer was deposited with a mass-equivalent thickness of 6 nm. The deposition was performed at rate of 0.07 nm s^−1^ and working pressure of 10^−5^ mbar. To guarantee the correct thickness and growth rates of the films, a calibrated quartz crystal detector was used. 

Figure 10 represents a schematic of the production process of ZnO NR arrays decorated with Ag NPs on cardboard substrates. To understand the effect of the length and width of the ZnO NRs on the SERS signal, the Ag NPs were deposited onto the different aforementioned rod structures with distinct synthesis times and temperatures.

### 3.2. Characterization Techniques

The DSC measurements of the cardboard substrates were made with a simultaneous thermal analyser (TGA-DSC—STA 449 F3 Jupiter) from Netzsch-Geratebau GmnH (Selb, Germany). In these experiments, ~5 mg of the cardboard substrates was placed into an open aluminium crucible and heated up to 550 °C in air at a heating rate of 5 °C min^−1^.

The ZnO NR crystallinity was determined by XRD using a PANalytical’s X’Pert PRO MRD (Almero, The Netherlands) X-ray diffractometer with a monochromatic CuKα radiation source (wavelength 1.540598 Å). The XRD measurements were made from 10° to 90° (2θ; and in a higher magnification from 30° to 40°), with a scanning step size of 0.016°. The ZnO nanocrystals were observed by SEM using a Carl Zeiss AURIGA CrossBeam Workstation (Oberkochen, Germany) instrument equipped with an Oxford X-ray EDS (Oberkochen, Germany).

Diffuse reflectance measurements of the samples were made at room temperature using a Perkin Elmer lambda 950 UV/VIS/NIR (Waltham, MA, USA) spectrophotometer with a diffuse light detection module (150 mm diameter integrating sphere, internally coated with Spectralon). The calibration of the system was performed by using a standard reflector sample (reflectance, *R*, of 1.00 from Spectralon disk). The reflectance (*R*) spectra was acquired from 250 to 800 nm.

R6G was chosen as a model analyte to test the efficiency of using ZnO NRs on the cardboard substrate, decorated by Ag NPs, as a platform in SERS applications. R6G is one of the preferential benchmark analytes, as it has been extensively studied and characterized by SERS [7,83]. A 2 µL volume of R6G solution with a concentration of 10^−6^ M was drop cast on the samples, and the Raman measurements were made before the drop dried. For the Raman measurements, a Labram 300 Jobin Yvon from Horiba (Kyoto, Japan) spectrometer equipped with an air-cooled CCD (charge-coupled device) detector was employed. For these experiments taken at room temperature, a HeNe laser was used at 17 mW and 632.81 nm. The data were recorded as an extended scan, and triplicates were taken of all spectra. The laser power at the surface of the analyzed material was fixed with the aid of a neutral density filter (optical density of 0.3, corresponding to a laser power of 660 µW). The measurements were made with five laser exposure scans of 25 s each, thus reducing the random detector background noise. A silicon wafer vibrational line of 521 cm^−1^ was used between the Raman measurements for calibration, thus avoiding possible system fluctuations. 

The uniformity of the Raman signal from the best-performing substrate (*t*_NR_ = 5 min, covered with 6 nm Ag mass thickness) was characterized from six randomly selected spots on the sample surface (2.5 × 2.5 cm^2^ area), separated by a distance of at least 1 cm. It was verified that the Raman spectra intensities from the different spots were almost identical, indicating a good uniformity (see Section S2 in Supplementary Information). In addition, the stability of the materials was tested by storing the samples for 6 months, which showed that the Raman peaks’ profile was similar to that of the newly prepared samples, suggesting rather stable SERS substrates.

## 4. Conclusions

In this work, ZnO NRs were synthesized on cardboard substrates by an innovative ultrafast method based on hydrothermal synthesis and assisted by microwave radiation. A set of synthesis times (5–30 min) and temperatures (70–130 °C) were tested. As the synthesis time and temperature increase, the diameter and length of the resulting ZnO NRs also increases. Their material band gap decreases with the increase in time and temperature, suggesting an increase in crystallite size. With the deposition of Ag NPs over the ZnO NRs, it was possible to test the produced substrates as platforms for SERS application, obtaining a remarkable average EF value of 7 × 10^5^, with the NR scaffold produced at 70 °C for 5 min.

The results presented here show that it is possible to grow well-aligned and highly crystalline ZnO NRs on cardboard-type substrates using an ultra-fast method, and that it is possible to use this as a low-cost, disposable platform for SERS detection in chemical and biological analytical devices.

## Figures and Tables

**Table 1:** Optical band gap of ZnO nanorods, produced with different synthesis time and temperature, obtained by extrapolating (*αhν*)^2^ vs *hν*.

Synthesis Time	Seed Layer	5 Min	10 Min	20 Min	30 Min
70 °C	3.275 eV	3.275 eV	3.265 eV	3.242 eV	3.240 eV
**Synthesis Temperature**	**Seed Layer**	**70 °C**	**90 °C**	**110 °C**	**130 °C**
30 min	3.275 eV	3.240 eV	3.242 eV	3.241 eV	3.241 eV

## Supplementary Materials

The following are available online at www.mdpi.com/1996-1944/10/12/1351/s1: Figure S1: (a) Differential scanning calorimetry (DSC) and (b) X-ray diffraction (XRD) diffractogram of cardboard substrate; (c) schematic of cardboard layered composition. Figure S2: Intensities of the 1360 cm^−1^ Raman vibrational lines of the spectra of 10^−6^ M R6G, acquired from the best-performing substrate (*tNR* = 5 min, covered with 6 nm Ag mass thickness) at six randomly selected spots on its surface. Each bar corresponds to the average from five individual spectra measured within the vicinity of each spot.
