# Supplementary material for: 3D ZnO/Ag Surface-Enhanced Raman Scattering on Disposable and Flexible Cardboard Platforms
Source: Materials (Basel). 2017 Nov 24;10(12):1351. doi: 10.3390/ma10121351 (PMC5744286; doi:10.3390/ma10121351)
Supplement: Supplementary file 1 [file materials-10-01351-s001.pdf]

3-D ZnO/Ag Surface-Enhanced Raman Scattering on Disposable and Flexible Cardboard Platforms

A. Pimentel, A. Araújo, B. Coelho, D. Nunes, M. J. Oliveira, M. J. Mendes, H. Águas, R. Martins, E. Fortunato

i3N/CENIMAT, Department of Materials Science, Faculty of Science and Technology, Universidade NOVA de Lisboa, Campus de Caparica, 2829-516 Caparica, Portugal;

S1- Thermal analysis and XRD of cardboard substrates

The DSC analysis of figure S1.a shows that the cardboard substrate starts to degrade at a temperature of 220 °C.

The XRD diffractogram obtained for the cardboard substrate (figure S1.b) reveals the peaks of cellulosic fibres at 2θ equal to 15°, 22° and 35°, corresponding to the crystallographic planes (110), (200) and (004). It can also be observed 3 peaks corresponding to the aluminium crystallographic phase, at 45°, 65° and 78°, associated to the crystallographic planes (200), (220) and (311), respectively.

The schematic on figure S1.c shows the different layers present on cardboard packaging substrates, consisting in cellulose fibres, polymeric coatings (polyethylene) and evaporated aluminium.

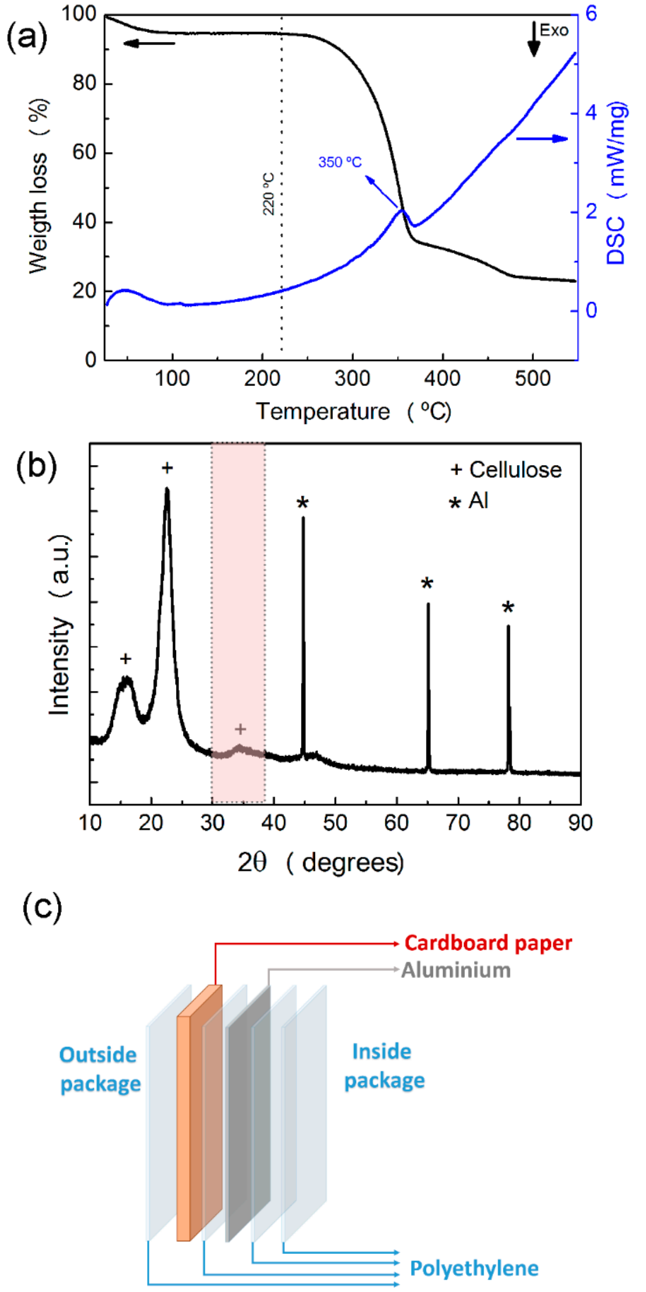

Figure S1: (a) Differential Scanning calorimetry (DSC) and (b) XRD diffractogram of cardboard substrate; (c) schematic of cardboard layered composition.

S2– Analysis of SERS substrates uniformity

To test the uniformity of the best-performing substrate (t_{NR} = 5 min, covered with 6 nm Ag mass thickness), additional SERS spectra of 10^{-6} M R6G molecules were collected from 6 randomly-selected spots on the same substrate (2.5 x 2.5 cm^2 area) separated by a distance of at least 1 cm. The Raman spectra profiles revealed to be quite similar, indicating high uniformity of the Ag NPs@ZnO NRs substrates and good reproducibility of the SERS signal.

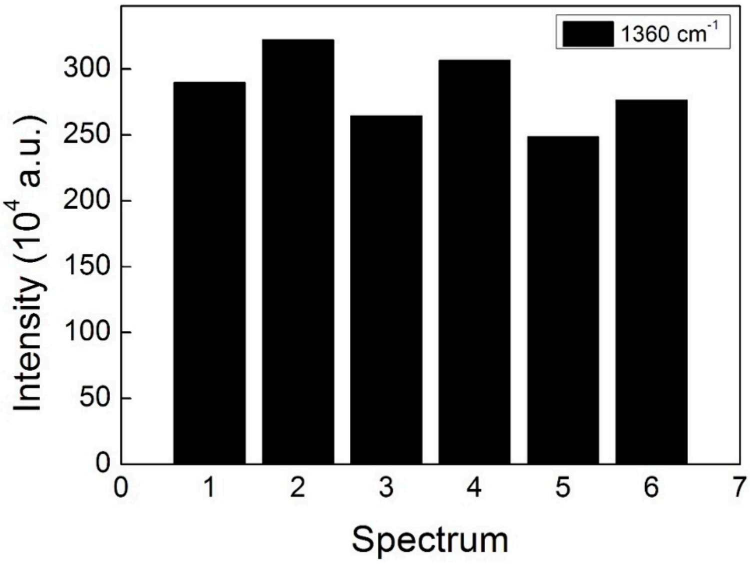

Figure S2 Intensities of the 1360 cm^{-1} Raman vibrational lines of the spectra of 10^{-6} M R6G, acquired from the best-performing substrate (t_{NR} = 5 min, covered with 6 nm Ag mass thickness) at 6 randomly selected spots on its surface. Each bar corresponds to the average from five individual spectra measured within the vicinity of each spot.
